# Learning from crowds in digital pathology using scalable variational Gaussian processes

**DOI:** 10.1038/s41598-021-90821-3

**Published:** 2021-06-02

**Authors:** Miguel López-Pérez, Mohamed Amgad, Pablo Morales-Álvarez, Pablo Ruiz, Lee A. D. Cooper, Rafael Molina, Aggelos K. Katsaggelos

**Affiliations:** 1 Department of Computer Science and Artificial Intelligence, University of Granada, 18071 Granada, Spain; 2 Department of Pathology at Northwestern University, Chicago, IL 60611 USA; 3 Microsoft Research, Cambridge, CB12FB UK; 4 OriGen.AI, Brooklyn, NY 11201 USA; 5 Department of Electrical and Computer Engineering at Nothwestern University, Evanston, IL 60208 USA; 6 Center for Computational Imaging and Signal Analytics, Northwestern University, Chicago, IL 60611 USA

**Keywords:** Cancer, Breast cancer

## Abstract

The volume of labeled data is often the primary determinant of success in developing machine learning algorithms. This has increased interest in methods for leveraging crowds to scale data labeling efforts, and methods to learn from noisy crowd-sourced labels. The need to scale labeling is acute but particularly challenging in medical applications like pathology, due to the expertise required to generate quality labels and the limited availability of qualified experts. In this paper we investigate the application of Scalable Variational Gaussian Processes for Crowdsourcing (SVGPCR) in digital pathology. We compare SVGPCR with other crowdsourcing methods using a large multi-rater dataset where pathologists, pathology residents, and medical students annotated tissue regions breast cancer. Our study shows that SVGPCR is competitive with equivalent methods trained using gold-standard pathologist generated labels, and that SVGPCR meets or exceeds the performance of other crowdsourcing methods based on deep learning. We also show how SVGPCR can effectively learn the class-conditional reliabilities of individual annotators and demonstrate that Gaussian-process classifiers have comparable performance to similar deep learning methods. These results suggest that SVGPCR can meaningfully engage non-experts in pathology labeling tasks, and that the class-conditional reliabilities estimated by SVGPCR may assist in matching annotators to tasks where they perform well.

## Introduction

The amount of labeled data is one of the primary determinants of performance in machine learning applications, and the requirements of today’s data-hungry algorithms have increased interest in scaling data labeling processes. A *crowdsourcing* approach that engages a broad set of individuals in labeling has been shown effective in tasks where expertise is not required such as labeling images in general categories^1–3^. In applications requiring expertise, sourcing labels from crowds is more challenging. Medical applications where labels are often assigned by expert diagnosticians with years of training are particularly difficult, but are arguably the applications where scaling is needed most due to the lack of availability of these experts and the clinical demands on their time^1,4,5^. Crowdsourcing in these scenarios can introduce significant tradeoffs between label volume and quality^4^. A more open process can generate more labels but may sacrifice quality. Engaging with more focused groups such as medical students that have some familiarity with the subject matter can improve quality and can enable some degree of vetting of participants.

Crowdsourced labeled data suffer from high label noise due to the different varying expertise degrees. One typical approach for obtaining reliable labeled data is the consensus, i.e., majority voting. However, in medical imaging, fixing/aggregating the noisy labels in a previous training step is not the best way. Instead, the best choice is to keep each annotation and model the expertise degree of each annotator. For example, weighting each annotation based on the annotator’s reliability achieves this purpose^6^. Raykar et al. introduced a crowdsourcing model for classification with multiple annotators^7^ based on logistic regression. This crowdsourcing framework jointly learns a latent classifier and annotators’ reliability. This model was used for grading prostate cancer in tissue microarrays^8^, where five different pathologists annotated each image. They estimated iteratively the classifier’s coefficients and the annotators’ reliability, following an Expectation–Maximization (EM) scheme. The logistic regression classifier overcame the inter-observer grading variability levels, and showed a good agreement with the participants. However, the flexibility of this model is limited, because it considers logistic regression as the latent classifier. An analogous crowdsourcing framework has been also used with more expressive classifiers such as deep neural networks^9,10^. Gaussian processes were also introduced for crowdsourcing with sound results across different domains^11–13^. These models are Bayesian and non-parametric, making them suitable to learn good models without the need for very large labeled datasets. Also, they provide an accurate estimation of the uncertainty in the predictions^14^.

In the dataset we will use in this paper, a group of medical students, pathology residents, and pathologists were organized to label tissue regions in digital pathology images of breast cancer specimens^15^. The average medical student may have some basic understanding of histology from their medical school coursework, but they will not have specific knowledge of histologic patterns in breast cancer^16^. The varied experience of these participants was leveraged to optimize effort while preserving quality. Medical students performed the majority of labeling tasks under the supervision of residents and attending pathologists, and feedback was provided openly via a Slack communication channel to avoid answering redundant questions. This significantly improved the quality of work that was given final review by pathologists, minimizing their work and interventions. While this process was effective, it worked because there was prior knowledge of participant experience, and it still required significant involvement of pathologists. This study set a high standard for quality for compatibility with learning algorithms that may not tolerate label noise well. A more tolerant algorithm would allow relaxation of these standards, enabling engagement of a broader audience without prior knowledge of their experience, and would require less oversight and review of their work. An ideal learning algorithm would be able to estimate the strengths and weaknesses of an individual participant during labeling, and to assign them examples accordingly to maximize efficiency^17^.

**Figure 1:**
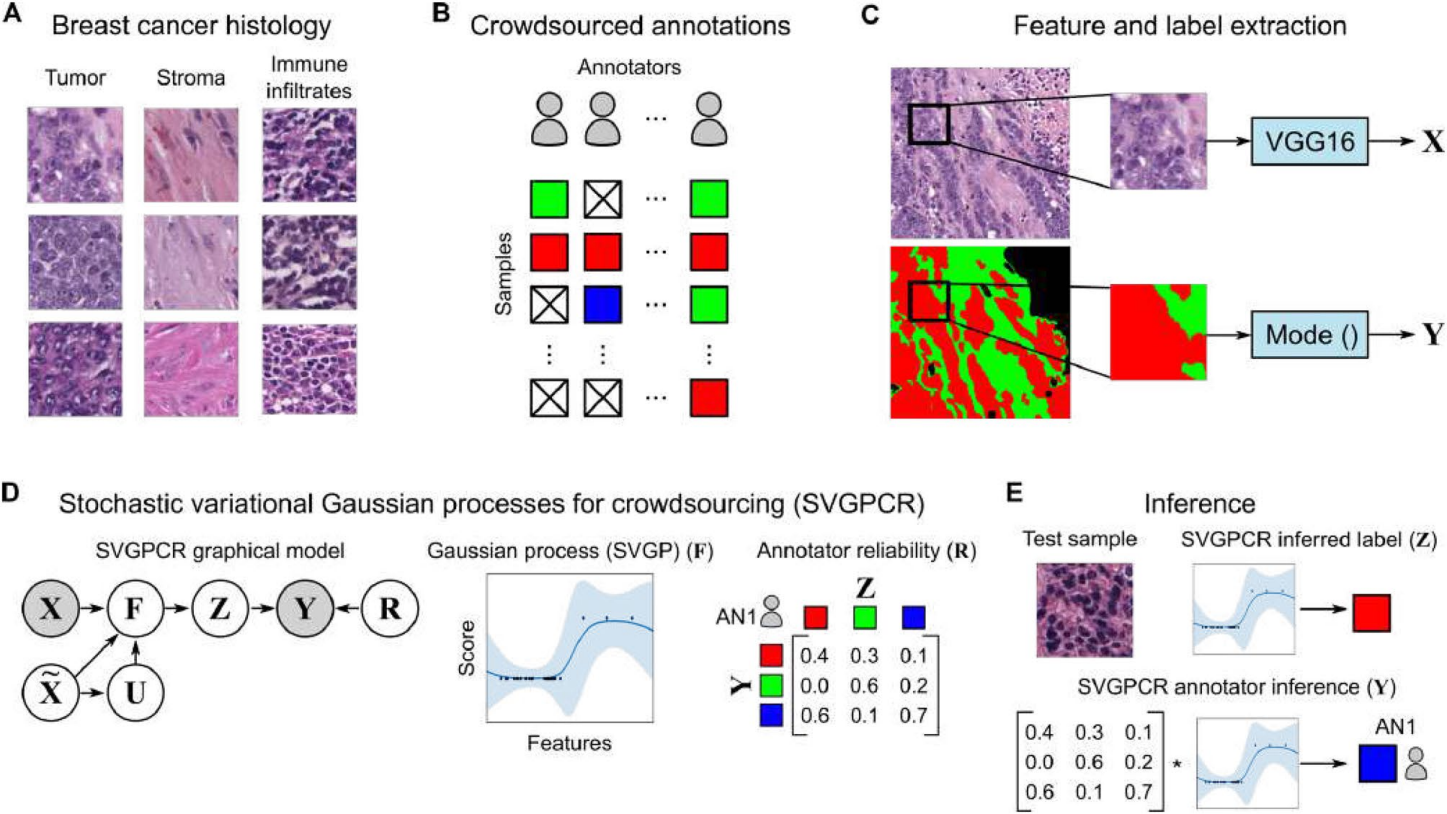
Scalable variational Gaussian processes for crowdsourcing (SVGPCR) in digital pathology. (**A**) This paper uses classification of predominant tissue patterns in breast cancer to investigate how SVGPCR can be used in crowdsourcing annotations for digital pathology. (**B**) The data used in this paper originates from a study where participants delineated tissue regions to produce semantic segmentation annotations in a set of curated Regions of Interest (ROI) (see Fig. 2). SVGPCR enables a sparse study where most ROIs are not annotated by all participants. (**C**) To leverage SVGPCR in this application, we analyze patches from the annotated ROIs. Patches were selected where at least 50% of the pixels correspond to a single label. For each patch with a majority label ${{\mathbf {Y}}}$ we used VGG16 to extract a 512-dimensional feature vector ${{\mathbf {X}}}$ for SVGPCR training. (**D**) In SVGPCR, the observed annotation ${{\mathbf {Y}}}$ depends on the true label ${{\mathbf {Z}}}$ and annotator reliability ${{\mathbf {R}}}$. The scalable variational Gaussian process (SVGP) classifier ${{\mathbf {F}}}$ is trained to predict the true label from the features ${{\mathbf {X}}}$. $\tilde{{{\mathbf {X}}}}$ and ${{\mathbf {U}}}={{\mathbf {F}}}(\tilde{{{\mathbf {X}}}})$ are used to improve the scalability of training (in GP terminology, they are called *inducing locations* and *inducing points* respectively, see Details on the machine learning algorithm). (**E**) Given a test patch, the SVGP classifier ${{\mathbf {F}}}$ can be used to infer the true label ${{\mathbf {Z}}}$, or combined with the reliability matrix of a specific annotator to infer how that annotator would label the patch.

In this paper we investigate how Scalable Gaussian Processes (SVGP) can learn from noisy crowdsourced labels in digital pathology applications (Fig. 1). We explore a previously developed technique, SVGP for Crowdsourcing (SVGPCR), that learns how to infer accurate labels by estimating class-conditional reliabilities for individual annotators^18^. SVGPCR can learn these reliabilities from sparsely annotated datasets where each sample is labeled by only a subset of the annotators. The probabilistic modeling used by SVGPCR is described in detail in Methods. We applied SVGPCR to a dataset where practicing pathologists, pathology residents, and medical students annotated breast cancer tissue regions. Our experiments found that SVGPCR trained on the noisy labels from non-experts is competitive with an equivalent SVGP trained using gold-standard expert labels. We also demonstrate how the learned annotator reliabilities accurately capture the class-conditional performance of individual annotators. We describe limitations of this approach and discuss how these approaches could be used to improve data labeling in digital pathology applications in the future. The code is publicly available at https://github.com/wizmik12/crowdsourcing-digital-pathology-GPs.

## Methods

**Figure 2:**
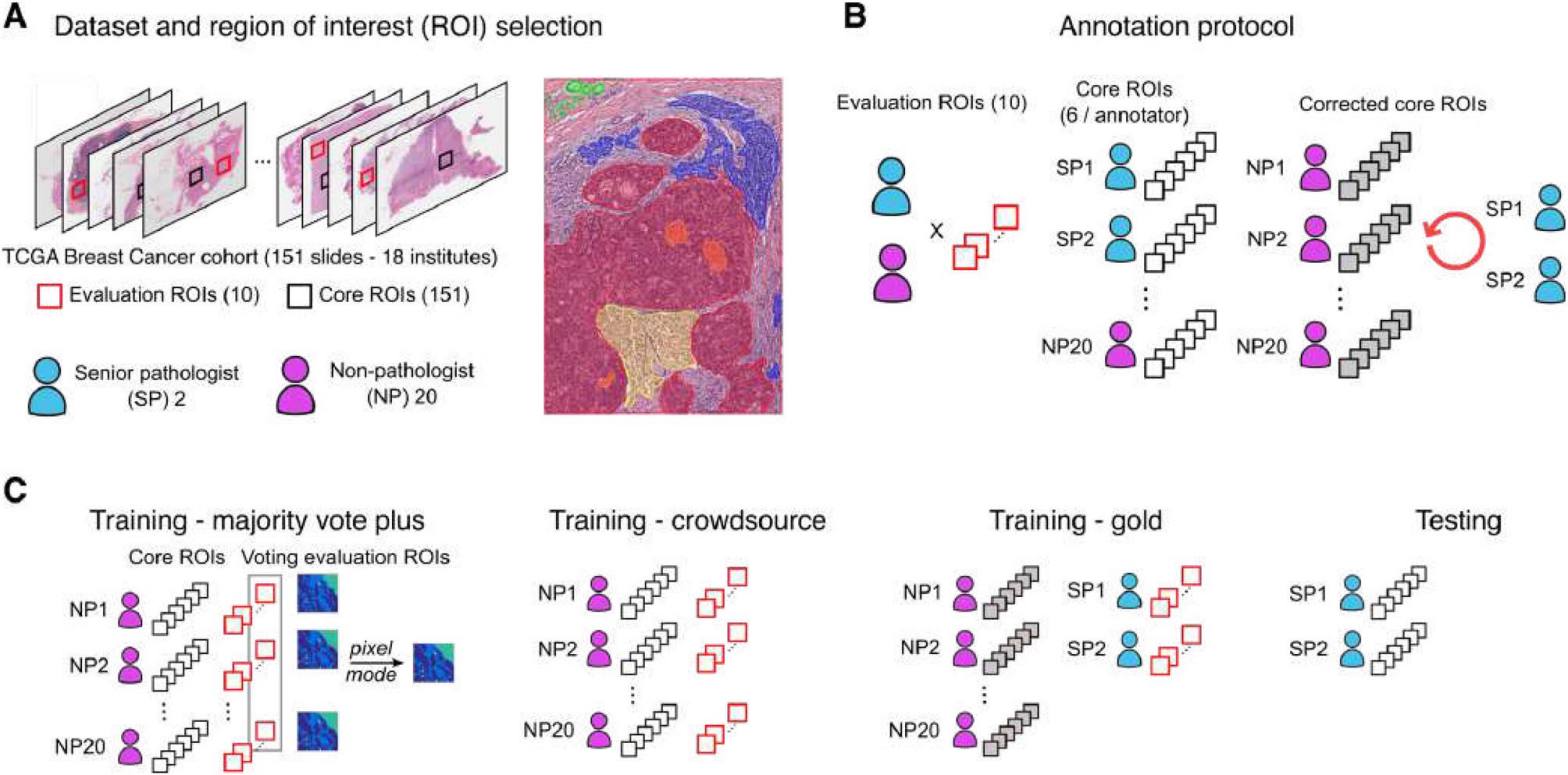
Experimental design. Our experiments combine annotations generated by experts (SP) and novice (NP) participants in a crowdsourcing study of breast cancer digital pathology images. (**A**) 161 regions of interest in 151 slides were selected for inclusion in the annotation study^15^. 10 ROIs were selected as the Evaluation ROIs (red) and annotated by all participants. The remaining 151 ROIs were each assigned to individual annotators as Core ROIs (black). (**B**) Participants used a web-based interface to annotate a number of tissue regions in each ROI including tumor, stroma, immune infiltration, and others. Core ROIs annotated by NPs were reviewed and corrected independently by either SPs, giving us paired uncorrected (black) and corrected gold standard (gray-filled) annotations. Annotations on Evaluation ROIs did not undergo correction. (**C**) We formed a number of training sets to assess various conditions. A “majority vote” (MV) training set smooths the labels over the evaluation set ROIs for assessing non-crowdsourcing methods. These are combined with the uncorrected core ROI annotations to increase data volume. A “crowdsource” (CR) dataset combines the uncorrected core and evaluation ROIs for NPs to form a training dataset with noisy labels for assessing crowdsourcing methods. A gold standard training dataset combines corrected ROIs from NPs with evaluation ROIs from the SPs. The testing set used to assess performance was composed of core ROIs from SPs and corrected core ROIs from NPs.

The data used in our experiments originate from an international study where pathology experts and non-experts annotated breast cancer tissue regions in a crowdsourcing process^15^. In this study a web-based platform was used to annotate breast cancer tissue regions by two senior/practicing pathologists (SP), and 20 non-pathologists (NP) consisting of medical students and fresh graduates. A study coordinator selected 161 rectangular regions of interest (ROIs) from 151 whole-slide images of formalin-fixed paraffin embedded sections from the TCGA Breast Cancer cohort. ROIs were selected to capture representative patterns of tumor, stroma, and immune infiltrates, as well as less common regions and structures including necrosis, blood vessels, and fat. Images and ROIs were hosted on a Digital Slide Archive server where participants could access them through a web-browser and use their mouse to annotate tissue regions in the ROIs using the polyline tool.

ROIs were assigned to two categories to provide both adequate breadth for training ML algorithms and to enable assessment of interobserver variability in annotation. Core ROIs provide breadth, being present in all 151 slides, and were divided among the users (approximately 6 per user) based on a difficulty score assigned by the study coordinator. Participants first annotated their core ROIs and then solicited feedback from an SP who applied corrections in multiple feedback cycles. This provided two versions of the core ROI: (1) Uncorrected core ROIs and (2) Corrected core ROIs. Ten additional Evaluation ROIs were created in the slide set and assigned to all NP participants to assess interobserver variability. Annotation of evaluation ROIs was performed following completion of core ROIs; evaluation ROI annotations were not corrected. The DICE coefficient for segmentation annotations made by SPs was as follows: 0.87 (tumor), 0.81 (stroma), and 0.52 (lymphocytic infiltration). Further details on the interobserver variability for both SPs and NPs is discussed in detail in Ref.^15^.

We performed a collection of experiments to assess the impact of training data quality and the effectiveness of crowdsourcing approaches. We considered a multiclass problem with three different classes: tumor, stroma, and immune infiltrates. We also compared Gaussian processes (with features from pre-trained convolutional networks) with state-of-the-art deep learning models like CrowdLayer^10,18^. Data quality was examined by formulating three training sets with varying label quality (see Fig. 2): (1) Gold standard training combines corrected core ROI annotations with SP annotations on evaluation ROIs; (2) Majority vote training (MV) combines uncorrected NP core ROI annotations with pixel-wise majority voting over NP evaluation ROI annotations; (3) Crowdsourcing training (CR) combines all uncorrected NP core ROI annotations and all NP evaluation ROI annotations. The gold standard training set represents a gold-standard where all annotations are generated, corrected, or approved by SPs. The MV training set represents a naive approach to improving data quality by averaging over noisy NP annotations. The CR training set represents a true crowdsourcing experiment where NP annotations are not corrected or revised by experts or smoothed through averaging.

First we measured the impact of training data quality on SVGP and VGG16 methods that weigh all labels and annotators equally, comparing their performance with smoothed label MV training and gold standard training. Next, we assessed the ability of crowdsourcing methods like AggNet^9^, CrowdLayer (CL)^10^, and SVGPCR^18^, which learn annotator reliability using CR training generated through crowdsourcing with non-experts. The first two are recent methods based on deep learning. For Crowdlayer, depending on the annotator modeling, we can distinguish three different models: CL-MW, CL-VW, and CL-VWB. CL-VW incorporates a vector of per-class weights, an additional bias is considered for CL-VWB and, the most complex, CL-MW computes the whole confusion matrix of the annotators. SVGPCR is based on scalable Gaussian Processes.

Finally, we assessed the ability of SVGPCR to infer predictions from a specific annotator that reflect that annotator’s class-conditional reliabilities. For these experiments we modified the CR training, reserving half of the evaluation ROIs for testing, and training the SVGPCR on the uncorrected NP core ROIs and the remaining evaluation ROIs. SVGPCR inference was performed for each annotator and evaluation ROI in the testing set and compared to the annotations of that annotator using the DICE coefficient. Dense predictions were generated in these experiments using sliding windows with 95% overlap to enable visual comparison.

**Figure 3:**
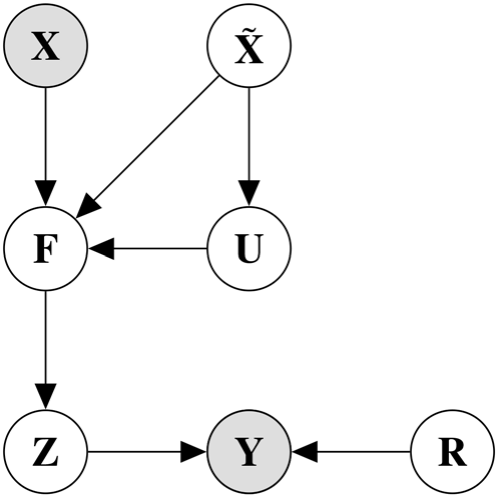
Probabilistic graphical model for SVGPCR. Dark variables refer to observed variables while light variables refer to latent variables (to be estimated). The observed variables are the features ${{\mathbf {X}}}$ and the annotations ${{\mathbf {Y}}}$ made by several annotators. The annotations depend on the true labels ${{\mathbf {Z}}}$ and the reliability of the annotators, ${{\mathbf {R}}}$. The true labels are modeled by latent variables ${{\mathbf {F}}}$ with a GP prior. Once the training is finished, the latent classifier can predict the true label on unseen samples. For scalability, $\tilde{{{\mathbf {X}}}}$ and ${{\mathbf {U}}}$ summarize data information lightening the computational cost ($\tilde{{{\mathbf {X}}}}$ is much smaller than ${{\mathbf {X}}}$).

Here we describe the formulation of a scalable SVGPCR algorithm that can learn from sparsely annotated datasets. Additional details are presented in the Supplementary Information and in the SVGPCR paper^18^. The inputs for training an SVGPCR model are the features ${{\mathbf {X}}}$, that are derived from the images, and the crowdsourced labels ${{\mathbf {Y}}}$. SVGPCR simultaneously learns both a classification model and the class-conditional reliabilities for each annotator. First, an underlying Gaussian Process (GP) model is learned to classify previously unobserved samples. The GP is denoted by ${{\mathbf {F}}}$ in Fig. 3 (${{\mathbf {U}}}$ and $\tilde{{\mathbf {X}}}$ are the inducing points and the inducing point locations respectively, and they are introduced for scalability). Second, the reliabilities of each annotator are modeled using per-annotator confusion matrices that describes the reliabilities of each annotator in labeling each class (${{\mathbf {R}}}$ in Fig. 3). Both ${{\mathbf {F}}}$ and ${{\mathbf {R}}}$ are connected by the variable ${{\mathbf {Z}}}$, which represents the unknown true labels of the training samples. This unknown variable is integrated out and estimated during training jointly with the classifier ${{\mathbf {F}}}$ and reliabilities ${{\mathbf {R}}}$.

This work addresses a *K*-class classification problem with crowdsourced labels. The training set consists of *N* instances $\{({{\mathbf {x}}}_n,{{\mathbf {y}}}_n^a):\; n=1,\dots ,N;\; a\in A_n\}$, where ${{\mathbf {x}}}_n\in {\mathbb {R}}^D$ is the feature vector of the *n*-th instance, and ${{\mathbf {y}}}_n^a$ is the label provided by the *a*-th annotator for the *n*-th instance. We represent labels as one-hot encoded vectors, i.e., the *k*-th class is specified by a vector in which all elements are zeros except for a single one in the *k*-th position. The matrix ${{\mathbf {X}}}=[{{\mathbf {x}}}_1,\ldots ,{{\mathbf {x}}}_N]^\intercal \in {\mathbb {R}}^{N \times D}$ contains the features of all the training instances and the set of all the annotations is defined as ${{\mathbf {Y}}}=\{{{\mathbf {y}}}_n^a: n=1,\ldots ,N, a\in A_n\}$ where $A_n$ is the subset of annotators that labeled the *n*-th instance. Note that each sample can be annotated by a different subset of annotators.

In this approach, each instance is assumed to have an (unknown) true label, ${{\mathbf {z}}}_n\in \{{{\mathbf {e}}}_1,\ldots ,{{\mathbf {e}}}_K\}$. The reliability of each annotator is modeled by a confusion matrix ${{\mathbf {R}}}^a=(r_{ij}^a)_{1\le i,j\le K}$. Each row of this matrix represents the label provided by the *a*-th annotator, and each column the true class. Notice that it is normalized, so each column adds up to 1, and the elements represent conditional probabilities. In other words, ${\mathrm {p}}({{\mathbf {y}}}^a={{\mathbf {e}}}_i|{{\mathbf {z}}}={{\mathbf {e}}}_j) = r^a_{ij}$. Notice that the reliability matrix of a perfect annotator will be the identity. Mathematically, this is given by

1 $$\begin{aligned} {\mathrm {p}}({{\mathbf {y}}}_n^a|{{\mathbf {z}}}_n,{{\mathbf {R}}}^a) = [{{\mathbf {y}}}_n^a]^\intercal {{\mathbf {R}}}^a {{\mathbf {z}}}_n. \end{aligned}$$

Assuming independence among annotators, we have

2 $$\begin{aligned} {\mathrm {p}}({{\mathbf {Y}}}|{{\mathbf {Z}}},{{\mathbf {R}}})= \prod _{n=1}^N\prod _{a\in A_n}{\mathrm {p}}({{\mathbf {y}}}_n^a|{{\mathbf {z}}}_n,{{\mathbf {R}}}^a), \end{aligned}$$

where ${{\mathbf {Z}}}=\{{{\mathbf {z}}}_n: n=1,\dots ,N\}$ and ${{\mathbf {R}}}=\{{{\mathbf {R}}}^a:a=1,\dots ,A\}$ contain the true labels of all instances and the reliability matrices of all annotators, respectively. model ${\mathrm {p}}({{\mathbf {y}}}_n^a|{{\mathbf {z}}}_n,{{\mathbf {R}}}^a)$ is the one defined in Eq. (1).

SVGPCR defines a prior (independent) Dirichlet distribution over ${{\mathbf {R}}}$,

3 $$\begin{aligned} {\mathrm {p}}({{\mathbf {R}}})= \prod _{a=1}^A\prod _{j=1}^K {\mathrm {p}}({{\mathbf {r}}}^a_j) = \prod _{a=1}^A\prod _{j=1}^K \mathrm {Dir}({{\mathbf {r}}}^a_j|\alpha _{1j}^a,\dots ,\alpha _{Kj}^a), \end{aligned}$$

where ${{\mathbf {r}}}^a_j=(r^a_{1j},\dots ,r^a_{Kj})^\intercal$ is the *j*-th column of ${{\mathbf {R}}}^a$. The hyperparameters ${\varvec{\alpha }}=\{\alpha _{ij}^a:i,j=1,\dots ,K,\; a=1,\dots ,A\}$ of the prior distribution allow for including assumptions on the reliability of the annotator. When there is no prior knowledge about the annotators’ behavior, the most common choice is to use a non-informative uniform distribution, i.e., $\alpha _{ij}^a=1$. senior peop. If this is not available, the default choice $\alpha _{ij}^a=1$.

So far, we have seen how SVGPCR models the crowdsourced annotations given the true labels. Now, we model the relationship between the true labels ${{\mathbf {Z}}}$ and the features ${{\mathbf {X}}}$ by introducing a latent classifier based on stochastic variational Gaussian procesess^19^. That is, *K* latent variables ${{\mathbf {f}}}_{n,:}=\{f_k({{\mathbf {x}}}_n)\}_{k=1}^K$ model the (unknown) true label ${{\mathbf {z}}}_n$ through a specific likelihood ${\mathrm {p}}({{\mathbf {z}}}_n|{{\mathbf {f}}}_{n,:})$. The latent variables provide scores in $\mathbb {R}$ to each sample and the likelihood maps them to the [0, 1] interval. likelihood plays a similar role as the output neurons play in DNNs. We use the soft-max likelihood which is defined by

4 $$\begin{aligned} {\mathrm {p}}({{\mathbf {z}}}_n={{\mathbf {e}}}_k|{{\mathbf {f}}}_{n,:})=\frac{e^{f_{n,k}}}{\sum _{c=1}^K e^{f_{n,c}}}. \end{aligned}$$

To lighten the notation, we denoted the latent variables by $f_k({{\mathbf {x}}}_n)=f_{n,k}$. Assuming that the class labels are independent given the latent variables, we factorize the likelihood across the different samples:

5 $$\begin{aligned} {\mathrm {p}}({{\mathbf {Z}}}|{{\mathbf {F}}})=\prod ^N_{n=1} {\mathrm {p}}({{\mathbf {z}}}_n|{{\mathbf {f}}}_{n,:}), \end{aligned}$$

where ${\mathrm {p}}({{\mathbf {z}}}_n|{{\mathbf {f}}}_{n,:})$ is given by Eq. (4). ${{\mathbf {F}}}$ gathers the latent variables in a $N\times K$ matrix where $f_{n,k}$ is placed in the *n*-th row and *k*-th column. Notice that the *K* latent variables are in the columns, ${{\mathbf {f}}}_{k}$, and the rows gather the value of each variable for the *N* instances ${{\mathbf {f}}}_{n,:}$.

The latent variables $\{{{\mathbf {f}}}_k\}_{k=1}^K$ are modeled by independent GP priors. This imposes that $\{f_{n,k}\}_{n=1}^N$ follow a multivariate Gaussian distribution (for a fixed *k*). We also assume that this Gaussian distribution has $\mathbf {0}$ mean and the covariance matrix is given by a kernel function. In this work, we use the Squared Exponential (SE) kernel, which is defined by $k({{\mathbf {x}}}_i,{{\mathbf {x}}}_j)=\sigma ^2\exp (-||{{\mathbf {x}}}_i-{{\mathbf {x}}}_j||^2/(2l^2))$^20^. Therefore, the prior over the latent variables ${{\mathbf {F}}}$ is given by

6 $$\begin{aligned} {\mathrm {p}}({{\mathbf {F}}}|{\varvec{\Theta }},{{\mathbf {X}}})=\prod ^K_{k=1}{\mathrm {p}}({{\mathbf {f}}}_k|{\varvec{\Theta }},{{\mathbf {X}}})=\prod ^{K}_{k=1}\mathcal {N}({{\mathbf {f}}}_k|\mathbf {0}, {{\mathbf {K}}}_{{{\mathbf {X}}}{{\mathbf {X}}}}), \end{aligned}$$

where ${\varvec{\Theta }}$ includes $\sigma$ and *l* (i.e., the kernel hyperparameters), and the covariance matrix is ${{\mathbf {K}}}_{{{\mathbf {X}}}{{\mathbf {X}}}}={{\mathbf {K}}}({{\mathbf {X}}},{{\mathbf {X}}})=(k({{\mathbf {x}}}_i,{{\mathbf {x}}}_j))_{i,j}$. Notice that the SE kernel is very expressive and performs remarkably well in different scenarios^20^. In particular, it encodes desirable properties in the covariance matrix, such as smoothness.

In summary, we have defined the following probabilistic model:

7 $$\begin{aligned} {\mathrm {p}}({{\mathbf {Y}}},{{\mathbf {Z}}},{{\mathbf {F}}},{{\mathbf {R}}}|{\varvec{\Theta }})= \underbrace{{\mathrm {p}}({{\mathbf {Y}}}|{{\mathbf {Z}}},{{\mathbf {R}}}){\mathrm {p}}({{\mathbf {R}}})}_{\text {CR modelling}} \underbrace{{\mathrm {p}}({{\mathbf {Z}}}|{{\mathbf {F}}})}_{\text {likelihood}}\underbrace{{\mathrm {p}}({{\mathbf {F}}}|{{\mathbf {X}}},{\varvec{\Theta }})}_{\text {GP prior}}. \end{aligned}$$

This model is not scalable because standard GPs involve the inversion of an $N\times N$ dimensional matrix. To overcome this limitation and deal with large datasets the sparse approximation is used^19^. This approximation introduces $M\ll N$ inducing points. These inducing points summarize the information of the observations and will lighten the computational cost. They are values of the GP function. Notice that the inducing locations, where the GP is valued to compute the inducing points, may not be instances of the training set. We denote by $\tilde{{\mathbf {X}}}=[\tilde{{\mathbf {x}}}_1,\dots ,\tilde{{\mathbf {x}}}_{M}]^\intercal \in \mathbb {R}^{M \times D}$ the inducing locations while ${{\mathbf {U}}}$ corresponds to their value after the GP is applied. In other words, ${{\mathbf {U}}}$ is the evaluation of the GP on $\tilde{{\mathbf {X}}}$, just like ${{\mathbf {F}}}$ is on ${{\mathbf {X}}}$. Importantly, the locations $\tilde{{\mathbf {X}}}$ are optimized during training. Finally, the sparse probabilistic model is given by

8 $$\begin{aligned} {\mathrm {p}}({{\mathbf {Y}}},{{\mathbf {Z}}},{{\mathbf {F}}}, {{\mathbf {U}}}, {{\mathbf {R}}}|{\varvec{\Theta }}) = \underbrace{{\mathrm {p}}({{\mathbf {Y}}}|{{\mathbf {Z}}},{{\mathbf {R}}}){\mathrm {p}}({{\mathbf {R}}})}_{\text {CR modelling}}\underbrace{{\mathrm {p}}({{\mathbf {Z}}}|{{\mathbf {F}}})}_{\text {likelihood}}\underbrace{{\mathrm {p}}({{\mathbf {F}}}|{{\mathbf {U}}},{\varvec{\Theta }}){\mathrm {p}}({{\mathbf {U}}}|{\varvec{\Theta }})}_{\text {GP prior}}. \end{aligned}$$

Once the probabilistic model is defined, the posterior distribution ${\mathrm {p}}({{\mathbf {Z}}},{{\mathbf {F}}}, {{\mathbf {U}}}, {{\mathbf {R}}}|{{\mathbf {Y}}},{\varvec{\Theta }})$ must be computed. Since this cannot be achieved in closed-form [integrating out ${{\mathbf {Z}}}$ in (8) is intractable], SVGPR resorts to variational inference. The mathematical details for the variational inference step and for the predictive distribution are provided in the Supplementary Information.

## Results

**Table 1:** Performance on the test set: F1 score, accuracy, log loss, and AUC values. Gold refers to expert labels, MV to majority vote labels, SVGPCR to crowdsource labels.

	F1 score	Accuracy	Log loss	AUC
VGG-gold	0.8088	0.8440	0.7073	0.9271
VGG-MV	0.7975	0.8325	0.6635	0.9201
SVGP-gold	0.8157	0.8582	0.3938	0.9373
SVGP-MV	0.7919	0.8458	0.4261	0.9289
SVGPCR	0.8147	0.8579	0.3983	0.9360

**Table 2:** Performance of crowdsourcing methods on the test set: F1 score, accuracy, log loss, and AUC values. These methods use non-expert labels.

	F1 score	Accuracy	Log loss	AUC
AggNet^9^	0.7998	0.8433	0.6814	0.9287
CL-MW^10^	0.8158	0.8570	0.4963	0.9317
CL-VW^10^	0.8072	0.8421	0.4911	0.9264
CL-VWB^10^	0.8179	0.8554	0.5536	0.9301
SVGPCR^18^	0.8147	0.8579	0.3983	0.9360

Table 1 depicts the performance of the SVGP and VGG methods with the different training sets. We found that training data quality impacts the performance of the SVGP and VGG methods. Training on the gold standard data resulted in improvements in F1 score, AUC, and accuracy for both SVGP and VGG when compared with MV training. For SVGP the gold standard training data improved the F1 score by 3.0% to 0.816. Similar improvements were observed for AUC (0.9% increase to 0.973), and accuracy (1.5% increase to 0.858). For VGG the gold standard training data improved the F1 score by 1.4% to 0.809. Similar improvements were observed for AUC (0.7% increase to 0.927), and accuracy (1.3% increase to 0.844). For log loss we observed an improvement for SVGP (7.6% reduction to 0.3938) but for VGG the loss increased (6.5% increase to 0.7073). Comparing SVGP and VGG with gold standard training we observed a small performance benefit for SVGP with a slightly higher F1 score (0.8% increase), AUC (1.0% increase), accuracy (1.7% increase), and lower loss (44% reduction) than VGG.

Table 2 depicts the performance of different crowdsourcing methods trained with the CR training set. CrowdLayer and SVGPCR have similar performance, with SVPGCR having a slight advantage in AUC, accuracy, and loss. CrowdLayer-VWB had a small advantage in F1 score (0.4% increase to 0.818), where SVGPCR had an advantage over the next best CrowdLayer method in AUC (0.4% higher than CL-MW), accuracy (0.1% higher than CL-MW), and loss (18.9% lower than CL-MW). AggNet has the lowest performance of crowdsourcing methods in all metrics except for accuracy. The best performing crowdsourcing methods were competitive with SVGP and VGG with gold standard training. SVGPCR trained on noisy CR labels is very similar to SVGP trained with gold standard labels with both methods having similar F1 scores (0.815 versus 0.816), AUCs (0.936 versus 0.937), accuracies (0.858 for both), and losses (0.398 versus 0.393). These differences are small when compared to differences between SVGP with MV training and SVGP with gold standard training.

**Figure 4:**
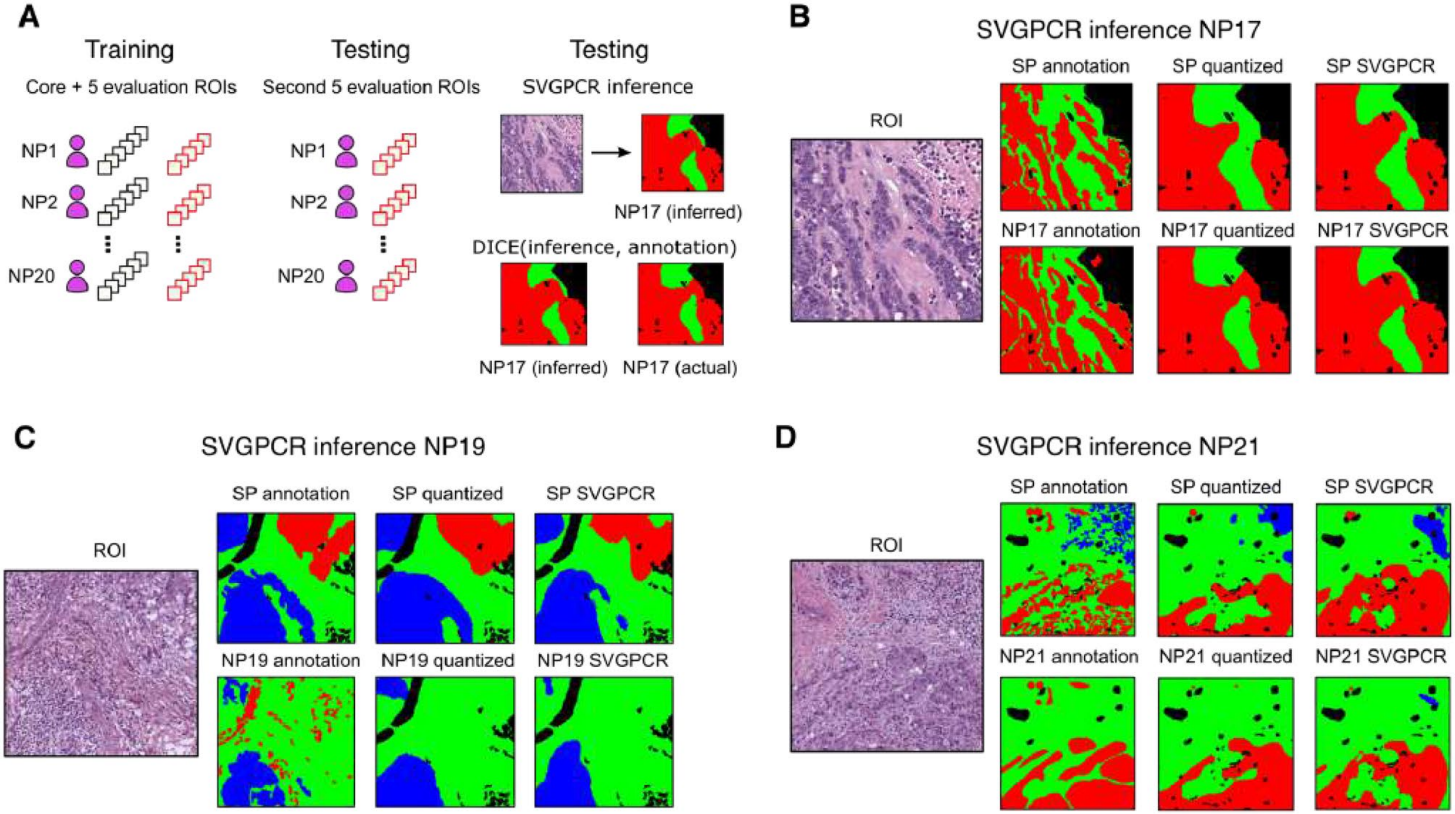
Visualizing annotator-specific inferences. We performed additional experiments to assess the ability of SVGPCR to learn the biases of individuals. The color in the masks encode tumor (red), stroma (green), lymphocytic infiltrates (blue) and other classes (black). (**A**) Two SVGPCR classifiers were trained. The first training set combined the core ROIs and first 5 evaluation ROIs, and performed inference on the second 5 evaluation ROIs. The evaluation ROIs were then swapped, and the training and inference were repeated. For each ROI, the trained SVGP and reliability annotation matrices were used to generate an annotator-specific inference. This inference was compared with the actual annotation and the annotation from the SP to observe differences. The patch-based analysis resulted in some quantization, so the quantized and original annotations are both presented. (**B**) This ROI contains a band of stroma from the upper center to the lower right that separates two regions of tumor, and a region of necrosis on the right. The inferred true labels correspond closely to the SP annotation. Participant NP17 is more sensitive in annotating tumor, and their inferred annotation exhibits the same pattern. (**C**) This ROI contains an island of tumor separated from regions of dense immune infiltrates by a wide area of stroma. The inferred true labels correspond closely with the SP annotation. Participant NP19 is not very sensitive in labeling tumor by comparison, and the tumor in the annotator inference is also absent. (**D**) This ROI contains tumor in the lower left and a small pocket of immune infiltrates in the upper right. The immune infiltrates are present in both the SP annotation and the inferred true labels. The immune infiltrates are absent from the annotation of participant NP21, and are mostly absent from the inferred annotation.

Figure 4 shows examples of inferred predictions for individual annotators. Visual inspection of these predictions shows that SVGPCR can learn and reproduce the biases of individual annotators. NP17 tends to call some stromal regions as tumor, and the SVGPCR inferred predictions for NP17 also exhibit this tendency. NP19 is less sensitive in annotating tumor, missing a large region that was annotated by the SP, and we see this same lack of sensitivity in SVGPCR inference for NP19. NP21 is not sensitive in detecting a group of inflammatory cells, and we also see that their SVGPCR inference lacks sensitivity in detecting these cells as well. Quantitative analysis of agreement between SVGPCR inferences for specific annotators and their uncorrected annotations is presented in Table 3. The quantization is made by reconstructing the pixel-level of annotators using the patches annotations. The similarity of the annotations and the predictions is performed using the DICE coefficient. This coefficient measures the similarity between them. The 95% confidence interval of the DICE scores averaged over the 20 NPs is $0.7789 \pm 0.0237$. The average DICE score when comparing SVGPCR inferred gold standard with the expert SP annotations lies outside this interval at 0.8072.

**Table 3:** DICE values for participant’s behavior and ground-truth (i.e., expert annotation) predictions. The results are computed per-class and globally. Furthermore, confidence intervals of 95% are computed for the 20 participants.

DICE	Tumor	Stroma	Immune infiltrates	Overall
Ground truth	0.8529	0.7979	0.6905	0.8072
Participant’s behavior	$0.8132 \pm 0.0342$	$0.7286 \pm 0.0392$	$0.4841 \pm 0.1310$	$0.7789 \pm 0.0237$

## Discussion

Data is often the limiting factor in training and validating machine learning algorithms for biomedical applications. When domain experts like pathologists are needed to produce ground-truth labels, generating data at the scale required by algorithms like convolutional networks is often difficult. This study seeks to address this problem by examining how a probabilistic approach to integrating annotations from novices can compete with algorithms trained using gold-standard data generated by experts. As a statistical machine learning method, Gaussian processes provide a framework for estimating the accuracy of annotators, including class-conditional accuracies, and to use this information in making inferences of ground truth. Our experiments show that SVGPCR trained on noisy labels obtained from novices in digital pathology crowdsourcing studies can compete with state of the art algorithms trained on gold standard labels.

We used a unique data resource to compare Gaussian processes based methods with other crowdsourcing approaches. The BRCA tissue region dataset contains over 20,000 tissue regions, including both novice and expert-corrected annotations, enabling comparison of crowdsourcing methods trained on novice annotations to methods trained on gold-standard annotations. Our experiments demonstrated that data quality impacts the performance of methods that are not based on crowdsourcing. SVGP and VGG models trained using a “majority vote” training dataset that averaged novice annotations had inferior performance compared to the same models trained using gold standard annotations. Under the optimistic conditions of training with gold standard annotations, SVGP and VGG had similar performance, with SVGP having a slight advantage in F1, AUC, accuracy and a large improvement in loss on the testing data, showing that Gaussian process models can compete with convolutional networks in this example.

The best crowdsourcing methods including SVGPCR and CrowdLayer variants trained using novice annotations have performance comparable to methods trained using gold standard annotations. This result suggests that in some circumstances, expert correction of novice annotations may not be necessary for annotations used in training. Performance differences for SVGPCR and CrowdLayer were small compared to differences between methods trained with majority vote and gold standard data, suggesting that the annotator and class conditional weighting applied by crowdsourcing methods is superior to basic smoothing of novice data labels. SVGPCR performance in classifying tumor and stroma was significantly higher than for immune infiltrates. This parallels the patterns of interobserver variability observed during the crowdsourcing study. Tumor and stroma are defined by sharp boundaries and in our annotation data we see significantly better concordance among annotators for these tissue types. Immune infiltration is diffuse and regions infiltrated by immune cells lack a sharp boundary, requiring annotators to judge their density which is much more subjective. This translates to higher interobserver variability among annotators for immune infiltrates, and likely presents a greater challenge for SVGPCR. Regions of immune infiltration are also less prevalent in our dataset than regions of tumor and stroma.

We also showed how SVGPCR can reproduce the biases of specific annotators through inference. This result suggests that SVGPCR could help assigning work to annotators on the basis of their relative strengths and weaknesses as observed in their class-conditional accuracies. By modeling class-conditional annotator accuracy, SVGPCR learns how to weight the labels of each annotator during training to improve inference of gold standard labels. We provide visual and quantitative evidence that show how annotator-specific inferences produced by SVGPCR agrees with the withheld annotations on these test images, and reflects the sensitivities of annotators to various classes.

While these results suggest that SVGPCR may help reduce the annotation burden in digital pathology tasks, there are some important limitations in our study. Quantizing segmentation annotations to the patch level was necessary to provide a neighborhood of pixels for SVGPCR to learn from, however, this results in a loss of detail. While this quantization was necessary to conduct our studies, SVGPCR may be more appropriate for patch level problems like cell classification than for segmentation problems where fine details need to be represented. While SVGPCR likely benefits from the presence of a variety of annotators, some being more specific or more sensitive for different classes, it is not well understood when variability in annotations may pose a problem for learning. Furthermore, while some common evaluations regions among annotators are likely necessary for SVGPCR to learn the strengths and weaknesses of each annotator, it is not well understood how the balance of evaluation and core ROIs impacts SVGPCR performance. The core regions increase the breadth of the training set, and the annotation of evaluation regions reduces this breadth given a fixed budget of annotator time. We also plan to explore how the class-conditional accuracies learned by SVGPCR can improve assignment data to participants in crowdsourcing experiments and can help participants to understand their weaknesses and to improve them. This could be accomplished by iterative training of an SVGPCR model during crowdsourcing studies. We are also interested in exploring how the number of evaluation and core regions impacts SVGPCR performance.

## Supplementary Information

Supplementary Information.
