# Supplementary material for: Learning from crowds in digital pathology using scalable variational Gaussian processes
Source: Sci Rep. 2021 Jun 2;11:11612. doi: 10.1038/s41598-021-90821-3 (PMC8172863; doi:10.1038/s41598-021-90821-3)
Supplement: Supplementary file 1 — Supplementary Information. [file 41598_2021_90821_MOESM1_ESM.pdf]

Learning from crowds in digital pathology using scalable variational Gaussian processes

Miguel López-Pérez[1], Mohamed Amgad[2], Pablo Morales-Álvarez[3], Pablo Ruiz[4], Lee A. D. Cooper[2,5,6,*], Rafael Molina[1], and Aggelos K. Katsaggelos[5,6]

[1] *Department of Computer Science and Artificial Intelligence, University of Granada, 18010 Granada, Spain.*
[2] *Department of Pathology at Northwestern University, Chicago, IL, 60611 US.*
[3] *Microsoft Research, Cambridge, CB12FB, UK.*
[4] *OriGen.AI, Brooklyn, NY, 11201 US.*
[5] *Department of Electrical and Computer Engineering at Nothwestern University, Evanston, IL, 60208 US.*
[6] *Center for Computational Imaging and Signal Analytics, Northwestern University, Chicago, IL 60611.*
[*] *Corresponding author: lee.cooper@northwestern.edu*

Variational inference and predictions

As mentioned at the end of the main paper, SVGPCR uses scalable variational inference for GPs (SVGP) [1, 2]. The goal of variational inference is to approximate the true posterior distribution $\mathrm{p}(\mathbf{Z}, \mathbf{F}, \mathbf{U}, \mathbf{R}|\mathbf{y}, \boldsymbol{\Theta})$ by an approximation $\mathrm{q}(\mathbf{z}, \mathbf{F}, \mathbf{U}, \mathbf{R})$. It also allows to estimate the hyperparameters $\boldsymbol{\Theta}$ (in this case, these are the kernel parameters).

In this probabilistic framework, the evidence lower bound (ELBO) arises naturally as the loss function of the model:

$$\log \mathrm{p}(\mathbf{Y}|\boldsymbol{\Theta}) \geq \int \mathrm{q}(\mathbf{Z}, \mathbf{F}, \mathbf{U}, \mathbf{R}) \log \frac{\mathrm{p}(\mathbf{Y}, \mathbf{Z}, \mathbf{F}, \mathbf{U}, \mathbf{R}|\boldsymbol{\Theta})}{\mathrm{q}(\mathbf{Z}, \mathbf{F}, \mathbf{U}, \mathbf{R})} \mathrm{d}\mathbf{Z}\mathrm{d}\mathbf{U}\mathrm{d}\mathbf{F}\mathrm{d}\mathbf{R}. \tag{1}$$

The ELBO is a lower bound of this evidence, which is not tractable in this problem. SVGPCR assumes the following parametric form for q:

$$\mathrm{q}(\mathbf{Z}, \mathbf{F}, \mathbf{U}, \mathbf{R}) = \mathrm{q}(\mathbf{Z})\mathrm{q}(\mathbf{F}|\mathbf{U}, \boldsymbol{\Theta})\mathrm{q}(\mathbf{U})\mathrm{q}(\mathbf{R}), \tag{2}$$

$$\mathrm{q}(\mathbf{F}|\mathbf{U}, \boldsymbol{\Theta}) = \mathrm{p}(\mathbf{F}|\mathbf{U}, \boldsymbol{\Theta}), \tag{3}$$

$$\mathrm{q}(\mathbf{U}) = \prod_{k=1}^{K} \mathcal{N}(\mathbf{u}_k|\mathbf{m}_k, \mathbf{S}_k), \tag{4}$$

$$\mathrm{q}(\mathbf{Z}) = \prod_{n=1}^{N} \mathrm{q}(\mathbf{z}_n) = \prod_{n=1}^{N} \mathbf{z}_n^{\intercal}\mathbf{q}_n, \tag{5}$$

$$\mathrm{q}(\mathbf{R}) = \prod_{a=1}^{A}\prod_{k=1}^{K} \mathrm{q}(\mathbf{r}_k^a) = \prod_{a=1}^{A}\prod_{j=1}^{K} \mathrm{Dir}(\mathbf{r}_j^a|\tilde{\alpha}_{1j}^a, \ldots, \tilde{\alpha}_{Kj}^a), \tag{6}$$

and then substituting in the ELBO (see eq. (1)):

$$
\begin{aligned}
&\log \mathrm{p}(\mathbf{y}|\boldsymbol{\Theta}) \geq \\
&\mathbb{E}_{\mathrm{q}(\mathbf{Z})\mathrm{p}(\mathbf{F}|\mathbf{U})\mathrm{q}(\mathbf{U})\mathrm{q}(\mathbf{R})} \log \frac{\mathrm{p}(\mathbf{Y}|\mathbf{Z},\mathbf{R})\mathrm{p}(\mathbf{Z}|\mathbf{F})\cancel{\mathrm{p}(\mathbf{F}|\mathbf{U})}\mathrm{p}(\mathbf{U})\mathrm{p}(\mathbf{R})}{\mathrm{q}(\mathbf{Z})\cancel{\mathrm{p}(\mathbf{F}|\mathbf{U})}\mathrm{q}(\mathbf{U})\mathrm{q}(\mathbf{R})} \\
&= \sum_{n=1}^{N} \sum_{a\in A_n} \sum_{\mathbf{y}\in \mathbf{Y}_n^a} \sum_{k=1}^{K} q_{nk} \mathbb{E}_{\mathrm{q}(\mathbf{r}_k^a)} \left[\log \mathrm{p}(\mathbf{y}|\mathbf{e}_k, \mathbf{r}_k^a)\right] \\
&+ \sum_{n=1}^{N} \sum_{k=1}^{K} q_{nk} \mathbb{E}_{\mathrm{q}(\mathbf{f}_{n,:})} \left[\log \mathrm{p}(\mathbf{e}_k|\mathbf{f}_{n,:})\right] - \sum_{n=1}^{N} \sum_{k=1}^{K} q_{nk} \log q_{nk} \\
&- \sum_{k=1}^{K} \mathrm{KL}(\mathrm{q}(\mathbf{u}_k)||\mathrm{p}(\mathbf{u}_k)) - \sum_{a=1}^{A} \sum_{k=1}^{K} \mathrm{KL}(\mathrm{q}(\mathbf{r}_k^a)||\mathrm{p}(\mathbf{r}_k^a)),
\end{aligned} \tag{7}
$$

where KL denotes the Kullback-Leibler divergence. By maximizing the ELBO in eq. (7), we find the optimal kernel parameters $\boldsymbol{\Theta}$ and the variational parameters of q, $\{\mathbf{q}_n : n = 1, \ldots, N\}$, $\{\mathbf{m}_k, \mathbf{S}_k : k = 1, \ldots, K\}$, and $\{\tilde{\alpha}_{ij}^a : i, j = 1, \ldots, K;\ a = 1, \ldots, A\}$. Importantly, the ELBO factorizes over the instances. This allows to train using mini-batches, reducing the computational cost.

When the training is finished and, therefore, the ELBO is optimized, we use the learned hyperparameters and variational parameters to predict on an unseen test sample, $\mathbf{x}_*$. The predicted value of the latent variable $\mathbf{f}_*$ on this point $\mathbf{x}_*$ is given by

$$
\begin{aligned}
\mathrm{p}(f_{*,k}|\mathbf{x}_*, \mathbf{X}, \mathbf{Y}) &= \int \mathrm{p}(f_{*,k}|\mathbf{u}_k)\mathrm{p}(\mathbf{u}_k|\tilde{\boldsymbol{\Theta}})d\mathbf{u}_k \\
&\approx \mathbb{E}_{\mathrm{q}(\mathbf{u}_k)}\mathrm{p}(f_{*,k}|\mathbf{u}_k) \\
&= \mathcal{N}(f_{*,k}|\tilde{\mu}, \tilde{\sigma}^2).
\end{aligned} \tag{8}
$$

The mean and the variance of this Gaussian distribution are defined as follows:

$$
\tilde{\mu} = \mathbf{K}_{\mathbf{x}_*\tilde{\mathbf{X}}}\mathbf{K}_{\tilde{\mathbf{X}},\tilde{\mathbf{X}}}^{-1}\tilde{\mathbf{m}}_k, \tag{9}
$$

$$
\tilde{\sigma}^2 = k_{\mathbf{x}_*\mathbf{x}_*} + \mathbf{K}_{\mathbf{x}_*\tilde{\mathbf{X}}}\mathbf{K}_{\tilde{\mathbf{X}},\tilde{\mathbf{X}}}^{-1}(\tilde{\mathbf{S}}_k - \mathbf{K}_{\tilde{\mathbf{X}}\tilde{\mathbf{X}}})\mathbf{K}_{\tilde{\mathbf{X}},\tilde{\mathbf{X}}}^{-1}\mathbf{K}_{\tilde{\mathbf{X}}\mathbf{x}_*}. \tag{10}
$$

Finally, the predicted label is obtained using the predicted latent variables $\mathbf{f}_*$ as

$$
\mathrm{p}(\mathbf{z}_*) = \int \mathrm{p}(\mathbf{z}_*|\mathbf{f}_*)\mathrm{p}(\mathbf{f}_*)d\mathbf{f}_*. \tag{11}
$$

To compute this integral, we resort to standard Monte Carlo sampling methods [1].

Another interesting task in crowdsourcing is to predict participant annotations on this new test sample, $\mathbf{x}_*$. The probability that the a-th annotator labels $\mathbf{x}_*$ as class i is given by

$$
\mathrm{p}(\mathbf{y}_*^a = \mathbf{e}_i) = \sum_{j=1}^{K} \mathrm{p}(\mathbf{z}_* = \mathbf{e}_j)\mathrm{p}(\mathbf{y}_*^a = \mathbf{e}_i|\mathbf{z}_* = \mathbf{e}_j). \tag{12}
$$

Notice that in eq. (11) and eq. (12), we omitted the explicit dependence on the observed variables $\mathbf{x}_*$, $\mathbf{X}$ and $\mathbf{Y}$ for simplicity.

References

[1] J. Hensman, A. G. de G. Matthews, and Z. Ghahramani, "Scalable variational Gaussian process classification," in *Proceedings of the Eighteenth International Conference on Artificial Intelligence and Statistics, AISTATS 2015, San Diego, California, USA, May 9-12, 2015*, 2015.

[2] D. M. Blei, A. Kucukelbir, and J. D. McAuliffe, "Variational inference: A review for statisticians," *Journal of the American statistical Association*, vol. 112, no. 518, pp. 859–877, 2017.
